# Nutritional Behavior in European Countries during COVID-19 Pandemic—A Review

**DOI:** 10.3390/nu15153451

**Published:** 2023-08-04

**Authors:** Oskar Wojciech Wiśniewski, Bartłomiej Czyżniewski, Wioletta Żukiewicz-Sobczak, Magdalena Gibas-Dorna

**Affiliations:** 1 Department of Cardiology-Intensive Therapy and Internal Medicine, Poznan University of Medical Sciences, 49 Przybyszewskiego Street, 60-355 Poznan, Poland; 2 Department of Nutrition and Food, Faculty of Health Sciences, Calisia University, 62-800 Kalisz, Poland; wiola.zukiewiczsobczak@gmail.com; 3 Faculty of Medicine, Collegium Medicum, University of Zielona Gora, 28 Zyty Street, 65-046 Zielona Gora, Poland; bartlomiejczyzniewski@gmail.com; 4 Collegium Medicum, Institute of Health Sciences, University of Zielona Gora, 28 Zyty Street, 65-046 Zielona Gora, Poland

**Keywords:** COVID-19, diet, eating behavior, eating habits, nutrition, pandemic, physical activity, SARS-CoV-2

## Abstract

COVID-19 is highly linked with hyperinflammation and dysfunction of the immune cells. Studies have shown that adequate nutrition, a modifiable factor affecting immunity and limiting systemic inflammation, may play an adjunct role in combating the negative consequences of SARS-CoV-2 infection. Due to the global lockdown conditions, the COVID-19 pandemic has contributed, among others, to restrictions on fresh food availability and changes in lifestyle and eating behaviors. The aim of this paper was to review the data regarding eating habits in European countries within the general population of adults and some specific subpopulations, including obese, diabetic, and psychiatric patients, during the COVID-19 pandemic. The PubMed database and the official websites of medical organizations and associations were searched for the phrases “COVID” and “eating habits”. Papers regarding the pediatric population, non-European countries, presenting aggregated data from different countries worldwide, and reviews were excluded. During the COVID-19 pandemic, unhealthy lifestyles and eating behaviors were commonly reported. These included increased snacking, intake of caloric foods, such as sweets, pastries, and beverages, and a decline in physical activity. Data suggest that poor eating habits that create a positive energy balance have persisted over time as an additional post-COVID negative consequence.

## 1. Introduction

The outbreak of the COVID-19 pandemic has unquestionably changed the world. Rapidly, all the attention of the community was attracted to developing the most effective strategy to restrict the spread of SARS-CoV-2. Beyond recommendations to wear masks, use disinfectants, and keep their distance, many countries introduced lockdowns to flatten the curve of new infections and gain time for healthcare systems [1]. In parallel, intensified efforts were put into designing a specific vaccine and discovering drugs targeting SARS-CoV-2 replication. Although a few types of vaccine and antiviral medications have been invented [2,3,4,5,6,7,8], the COVID-19 pandemic has left an imprint on the global population. At the moment of writing, nearly 650 million COVID-19 cases have been diagnosed worldwide, while the total death toll has reached over 6.5 million people [9].

However, the impact of the COVID-19 pandemic goes much further and is not limited to the people summarized in the abovementioned statistics. A myriad of lifestyle changes referring to different areas of life have been observed. Increased levels of stress, social distancing, and household confinement are only a few of the contributing factors that fueled alterations in eating habits [10]. Since a well-balanced diet plays a primary role in the proper functioning of the immune system [11], particular attention has been paid to introducing healthy nutritional habits during the COVID-19 pandemic. For instance, it has been evidenced that only 10% of people consuming over 500 g of fruits and vegetables and more than 10 g of nuts daily have contracted COVID-19 infection, compared to 45% of those who ate less than 500 g of fruits and vegetables and less than 10 g of nuts daily [12]. As a poor diet is closely connected with increased inflammation and oxidative stress, dietary compounds showing anti-inflammatory, anti-oxidative, and immunomodulatory properties have been widely recommended during the COVID-19 pandemic (Table 1) [13]. Finally, the clinical significance of gut microbiota dysregulation in COVID-19 infection has been recently emphasized [14,15]. Hence, many research groups aimed to establish dietary guidelines to minimize the populational risk of acquiring COVID-19 infection or prevent severe-course COVID-19 [16,17,18,19].

Recommendations for the general population during the COVID-19 pandemic highlight the magnitude of a regular, well-balanced diet and encourage the consumption of a varied diet rich in fresh, unprocessed food, vegetables and fruits (at least four to five servings a day), wholegrain products, and healthy fats (linseed oil, olive oil, and fish oil), and drinking enough water, according to water requirements depending on age, sex, level of physical activity, diet, body composition, pregnancy, and environmental conditions [17,18]. It is also advised to limit intakes of red meat, sweet drinks, and food high in calories and salt. Supplements use is recommended for individuals with vitamin and/or micronutrient deficiencies [17,18]. Further studies and clinical trials are needed to prove the significance of vitamins and trace element supplementation in high-risk groups for COVID-19.

The aim of this narrative review is to provide a synthesis of the data regarding changes in eating habits during the COVID-19 pandemic reported in distinct European countries. In addition, we presented alterations in physical activity and sleep duration included in the cited research to show a broader context of lifestyle changes affecting body weight and populational health.

## 2. Methods

On 1 August 2022, the PubMed database was searched with the term “COVID AND eating habits”, which translated to the far-reaching query (“COVID-19” [Supplementary Concept] OR “COVID-19” [All Fields] OR “COVID” [All Fields] OR “SARS-CoV-2” [MeSH Terms] OR “SARS-CoV-2” [All Fields] OR “COVID-19” [MeSH Terms]) AND (“feeding behavior” [MeSH Terms] OR (“feeding” [All Fields] AND “behavior” [All Fields]) OR “feeding behavior” [All Fields] OR (“eating” [All Fields] AND “habits” [All Fields]) OR “eating habits” [All Fields]”). In addition, we explored several websites of medical organizations and associations, e.g., the British Nutrition Foundation, which helped to identify one of the relevant publications. Initially, 1194 articles were found. In the next step, the whole list of 1194 publications was carefully screened by two independent researchers, who decided if the paper was relevant to include in our review based on the titles. By comparing their results, a list of 422 articles for further evaluation was created. One doubled record was deleted when checking the correctness of the list. Since the number of papers significantly exceeded the capacity of one review manuscript, we decided to focus on publications from European countries. Thus, we first excluded articles from non-European countries, presenting aggregated data and reviews from different countries worldwide. As a result, 188 studies were subjected to further assessment. Due to there still being a lot of studies, publications regarding the pediatric population were also excluded. Then, the remaining 154 papers were assessed for eligibility. By analyzing abstracts and full texts, 17 publications were found irrelevant to the topic of this review. Moreover, ten articles were excluded since they presented aggregated data from different European countries. Since the associations between COVID-19 and eating habits have already been extensively studied in numerous Mediterranean countries and summarized in a recent systematic review by Mignogna et al. [84], we also decided not to include the 64 papers from this region in our analysis of the general population. Thus, our review compiles the results of 49 observational studies performed in 14 European countries within the general population of adults, as well as 14 research papers focusing on specific subpopulations, including obese, diabetic, and psychiatric patients (Table 2).

## 3. General Population of Adults

### 3.1. Central and Eastern European Countries

Among 24 relevant papers from the region, the vast majority (18) referred to the Polish population. Most studies evidenced that about 40–50% of the participants ate more during the COVID-19 pandemic and admitted more frequent snacking [85,86,87,88,89]. This pattern was even better expressed in overweight and obese subpopulations, indicating that fixed unhealthy behavior is easier to aggravate [85,89,90]. Similarly, a higher increase in alcohol consumption was observed in alcohol addicts than in the general population (64% vs. 14%), and over 45% of smokers reported smoking more [85]. Thus, special attention should be paid to lifestyle changes in the most susceptible groups characterized by sustained unhealthy habits.

Furthermore, the daily number of meals was significantly elevated during the first wave of the COVID-19 pandemic [87,91], while no alterations were found six months after the pandemic outbreak [92]. As for dietary composition, most studies revealed that consumption of fast-food products, instant soups, sweet beverages, and energy drinks decreased, while a concomitant increase in sweets, eggs, potatoes, canned meat, and alcohol was noted [87,89,91,93]. Contrarily, reduced or unchanged alcohol use was reported in a few studies [89,94,95,96,97]. Moreover, elevated consumption of water, fruits, vegetables, and nuts was reported in a group of Polish primary school teachers [94] and Serbian students [95]. Notably, the most profound changes in the structure of consumed food were observed in the individuals who feared COVID-19 infection and strictly followed the isolation rules [98]. Interestingly, the vast majority of the respondents did not notice limited food availability, while it affected over 40% of those who changed their diet to be more unhealthy during the COVID-19 pandemic [88]. Furthermore, dietary guideline adherence appeared to be the lowest in people not coping well with stress and mental load, showing that this subgroup is the most predisposed to nutritional behavior deterioration during the COVID-19 pandemic [99]. Problematic eating behavior was diagnosed in over 14% of Polish adults, according to the Yale Food Addiction Scale 2.0 [100]. In addition, a few changes associated with education level were observed. Approximately one-third of the participants with higher education diplomas surveyed over a year after the first COVID-19 case in Poland reported eating more sweets than before the pandemic outbreak, indicating that dietary changes described during the first lockdown were mostly maintained [101].

Moreover, decreased physical activity reported by around 40–50% of the participants was a common finding [85,88,96,100,101]. Quantitatively, the mean metabolic equivalent of tasks (MET) declined by 30% during the first lockdown, while the mean value of sitting time elevated by 25% [93]. As a result, 30–50% of the individuals gained weight [85,86,87,89,90,92,96,100,101]. A mean increase in body weight varied from 0.5 kg to 6.5 kg [85,86,87,91,100], depending on the published paper. In addition, sleep quality diminished significantly [93,102]. Intriguingly, contrasting results were obtained in a broad Lithuanian research in which no changes in BMI and shortening of sleep time only in women aged 26 or more were noted [97]. However, a stable BMI has to be interpreted along with the fact that, during the COVID-19 pandemic, people tended to overeat less, according to the study, despite a significant decline in total MET observed.

Interestingly, the outbreak of the COVID-19 pandemic resulted in an increase in the consumption of foods that are believed to modulate immune responses, such as garlic, ginger, honey, lemon, raspberry syrup, turmeric, and fermented fruits and vegetables [103]. Additionally, the intake of dietary supplements containing vitamins C and D, zinc, and omega-3 fatty acids was elevated during the first two waves of the pandemic, as nearly 35% of Polish adults started taking at least one new dietary supplement [103] and an increase in spending on supplements was declared by almost 23% of the participants [104]. Correspondingly, over 40% of Serbian medical students declared higher supplement use in general, especially vitamin C, D, and zinc-containing products [95]. However, the opposite results were disclosed over a year after the COVID-19 pandemic outbreak in Poland, when no changes in the intake of vitamin C, D, and magnesium supplements were reported [101]. The possible explanation may be that the first fear of COVID-19 induced higher supplementation of immune modulators and receded over time, which was partially evidenced by Hamulka et al. [103], or that people felt safer after the vaccination program had started and reduced supplement use. Tangentially, people with appropriate nutritional knowledge were characterized by a higher pro-health diet index in March 2020, at the beginning of the pandemic, whereas no statistically significant association was found in October 2020, suggesting that people lacked the perseverance to uphold beneficial dietary habits introduced just after the first COVID-19 cases in Poland [92]. In this context, the fact that healthy eating apps were evidenced to encourage favorable behavior and healthy eating motives during the COVID-19 pandemic should not be underestimated [105].

Of note, the reported impact of the COVID-19 pandemic was even more expressed among university students. Over 50% of undergraduates declared that the pandemic changed their dietary habits adversely [106], while 72–76% stated it had a negative influence on their physical activity [106,107]. On the other hand, beneficial aftermaths of the COVID-19 pandemic involved 30% of university students for nutrition behavior and 20% for physical activity [106]. More optimistic data were unveiled by Hoffmann et al., who noted that nearly 20% of the surveyed (mostly young adults) reported a more healthy diet during the second wave of the COVID-19 pandemic in Poland, and 3% even ceased stimulants, whereas only 11% admitted a less healthy dietary pattern [104]. Similar results concerning an expanded population of Polish adults were observed by Górnicka et al., who showed that 27.6% changed their dietary regimen into a healthier one, while 19.4% turned to detrimental behaviors [88]. Additionally, a smaller percentage of the respondents (ca. 30%) decreased their physical activity than in other papers [104]. Furthermore, Szczepańska et al. revealed that not only did university students reduce the consumption of sweets, fast food, salty snacks, and energy drinks during the COVID-19 pandemic, but a slight tendency in improved breakfast regularity was also described [108].

### 3.2. Scandinavian Countries

Data from Scandinavian countries are rather scarce; only three relevant papers have been published so far [109,110,111]. Results from the Danish study confirmed that the lockdown policy led to increased food intake, and both more frequent snacking and cooking at home were responsible for the raised consumption [110]. Additionally, dietary habits changed negatively as lower intake of fruits and vegetables with a concomitant surge in pastries and alcohol consumption were noted. As a result of lockdown limitations, nearly 50% of individuals reduced their physical activity. Consequently, almost 30% of Danish participants declared weight gain. Furthermore, the Norway research endorsed that elevated use of high-sugar products during the COVID-19 pandemic may be partially explained by emotional eating, since the groups presenting with substantial worries or psychological distress reported consuming high-sugar foods and beverages much more frequently [111]. In addition, emotional eating, defined as eating more in response to feeling down or unsatisfied, was observed in up to 54% of Norwegian adults, affecting mainly women (62% females vs. 43% males). On the other hand, a Swedish study involving university students showed significant improvements in dietary habits, albeit the outcomes should be carefully analyzed in the context of the less restricted anti-COVID-19 policy compared to other countries [109]. Nonetheless, after three months of the pandemic (first follow-up), more students declared eating breakfast more frequently, while the intake of morning and afternoon snacks decreased. Moreover, a significant hike in daily physical activity and a decline in sitting hours took place. All these positive changes were additionally strengthened after the summer holidays (second follow-up).

### 3.3. Western European Countries

The negative influence of the COVID-19 pandemic was also observed in Western European countries. Approximately 25–30% of the participants from Germany admitted weight gain after the national lockdown, while only about 15–20% of the studied population lost weight concurrently [112,113,114]. Moreover, a significant increase in the consumption of sweets, pastries, bread, coffee, and alcohol was noted [112,113,114]. Contrarily, the amount of meat, fast food, and ready-made meals consumed declined substantially [112,113]. Interestingly, the elevated intake of confectioneries was recognized as the most associated with putting on weight [112,113]. Weight gain appeared more likely in women as well as in individuals with a higher baseline BMI and experiencing elevated mental stress [112]. Furthermore, decreased levels of general physical activity and more deteriorated sleep quality predominated during the lockdown and might have affected body weight [113]. Importantly, the introduction of lockdown policies contributed widely to eating behavior alterations, including eating culture and philosophy. Over 40% of German adults surveyed by Bühlmeier et al. indicated higher awareness of their diet composition and the significance of nutrition in maintaining health [114]. Furthermore, the majority of the participants confessed to trying new food or recipes, and almost 45% of them ate in a company or relished their favorite dishes more frequently [114]. Similarly, French studies evidenced that nearly half of the individuals paid attention to a healthy diet and changed their dietary habits [115]. Of note, reducing the intake of unhealthy food was slightly more prevalent than increasing the intake of wholesome products. Importantly, around two-thirds of people maintained dietary modifications established during lockdown for at least a few months after lockdown withdrawal [115]. Thus, changes in nutritional patterns initiated during the COVID-19 pandemic may still affect populational health, which is the best rationale for creating analyses like our paper.

Tangentially, almost 30% of French adults declared elevated daily food intake, and over 12% of them mentioned difficulties in controlling eating [116]. Correspondingly, nearly 30% of participants confirmed increased consumption of salty and caloric foods. Moreover, raised use of alcohol, cannabis, and tobacco was found in 25–30% of the surveyed patients. Augmented levels of stress and a decline in well-being were acknowledged as the principles for the introduction of unhealthy habits, and women were found to be more prone to such modifications [116]. Interestingly, the outcomes presented by Marty et al. showed that approximately half of the respondents were more susceptible to making their food choices depending on their current mood, which was closely connected with a declined nutritional quality [117]. Lowering the value of meals consumed was observed in the French population in general, with a higher intake of confectioneries, processed meat, and alcohol. On the other hand, over one-fourth of French adults paid more attention to health and weight control during the national lockdown, resulting in an increased nutrition value of the regular diet [117]. Additionally, more positive changes were reported by Sarda et al., who evidenced that the group of French adults undertaking beneficial diet modifications slightly predominated in the society [118]. More frequent home cooking and increased intake of fruits, vegetables, and fresh products, driven by the willingness to maintain health and weight, led to a more balanced diet. Contrarily, the rise in snacking, comfort food consumption, and difficulties in food availability were recognized as the causes of lowering the diet quality [118]. Finally, the percentage of university students suffering from eating disorders expanded substantially since the COVID-19 pandemic [119].

Surprisingly, data from the neighboring Benelux countries, including Belgium and the Netherlands, reveal that inhabitants were the most invulnerable to changing their dietary patterns during the COVID-19 pandemic. Approximately 80% of Dutch adults did not alter their nutritional habits and diet composition [120,121]. In addition, the negative impact of the pandemic may be principally observed throughout other papers. Drieskens et al. demonstrated that almost 30% of the surveyed gained weight as soon as after six weeks of household confinement [122]. An even higher percentage of participants putting on weight was reported in both overweight and obese subgroups, reaching nearly 40% in the latter [122]. Correspondingly, obese individuals were more likely to worsen their dietary choices during the lockdown than people with a normal BMI [120]. Increased snacking was associated the most with weight gain, apart from elevated alcohol and sweet beverage consumption and restricted physical activity [122]. Interestingly, unfavorable dietary acts were caused mainly by unhealthy food temptations at home, boredom, and having more spare time [120]. Similar remarks adhere also to the population of Dutch seniors. Up to one in three reported snacking more, and nearly 50% admitted a decline in physical activity, which resulted in weight gain in over 30% of the participants [123]. Of note, eating less than usual, skipping warm meals, or losing weight were confessed to by about 10% of the elderly respondents, also showing an increased risk of malnutrition and frailty due to the COVID-19 pandemic [123]. Intriguingly, 10% of Belgians worried about food shortage issues and decreased the consumption of fresh fruits and vegetables [124]. Finally, an overall decline in self-assessed mental health status was evidenced in the population of Belgian adults [125].

Comparable observations were noted in the United Kingdom. Most of the research revealed a higher food consumption by approximately 40–50% of the patients [126,127,128]. Increased use of sugary drinks and intensified snacking (chocolate, cakes, biscuits, and crisps) were widely reported [126,129,130]. Almost one-third of the studied group gained weight [126]. Decreased physical activity was reported by ca. 50% of individuals and associated with low income [129]. Interestingly, 35% of adults confessed to elevated alcohol consumption associated with anxiety, stress, and tiredness during the first pandemic lockdown [130]. According to the other study, over half of the respondents declared eating control difficulties [131], while Robinson et al. disclosed that 49% of surveyed people fell into unhealthy eating habits, although 88% of them confirmed they had time to eat healthily and 79% could afford wholesome food [129]. In the study by Herle et al., 16% of participants presented persistently eating more, and loneliness was recognized as a risk factor [132]. Importantly, people with a higher BMI were much more vulnerable to introducing detrimental lifestyle changes due to the pandemic [129,132]. However, several beneficial alterations were also described in the literature. Coffee, vegetables, and bread appeared to be consumed more often during the COVID-19 pandemic, while a concomitant reduction in the intake of meat was observed, which was associated with lowering the risk of acquiring SARS-CoV-2 infection [133]. In addition, an increase in vegetable consumption was reported by 12% of British adults, particularly when bored, anxious, stressed, or tired [130]. Moreover, almost 20% of adults started eating more fruits [130]. Interestingly, over 85% of respondents acknowledged the importance of taking immune system-boosting supplements during the COVID-19 pandemic [127]. In addition, almost 80% of Britons declared they considered buying healthier food during the second wave of the COVID-19 pandemic and that the pandemic affected the amount of food purchased [127]. Additionally, Baceviciene et al. evidenced a significant decline in unhealthy eating habits and a lengthened sleep duration among university students during the lockdown period [131].

## 4. Specific Populations

Apart from the general population of adults, several studies regarding specific groups of patients have been published so far, including those suffering from obesity, diabetes mellitus, and psychiatric disorders. Due to their high prevalence, the insight into dietary habits alterations provoked by the COVID-19 pandemic may help to establish more personalized dietary recommendations and improve community health in the future.

Similar results were obtained in both obese and diabetic patients. All the cited papers reported elevated consumption of snacks, sugary, and packaged foods during the pandemic in both subpopulations [134,135,136,137]. Moreover, a higher number of daily meals and decreased physical activity were noted [134,135,137]. On the other hand, a few positive features have been observed, as the regularity of meals improved and people tended to prepare meals by themselves much more frequently [135]. Furthermore, a raised consumption of vegetables, nuts, and water was described [137]. Nevertheless, statistically significant weight gain was evidenced in all the studies, affecting 39% to almost 55% of the individuals [134,135], albeit some people also managed to lose weight, according to both Grabia et al. [135] and Yazici et al. [134]. Interestingly, a substantial increase in sleep duration was disclosed, which might be associated with enhanced stress levels, fear, and disturbed mental health [134].

In addition, significant changes have been observed in morbidly obese patients who underwent bariatric surgery [138,139]. Although 55% of the individuals had managed to maintain body weight a year after the surgery and had lost a mean of 30% of body weight, which indicates a good adherence, COVID-19 pandemic lockdowns resulted in approximately a 1.5% increase in body weight reported by almost 75% of patients [138]. This hike was expressed to an even greater degree in the subgroup that had contracted COVID-19 infection and women [138]. Similarly, a higher regain of body weight in the most vulnerable period after bariatric surgery was observed during the COVID-19 pandemic than before [139]. Additionally, lowered quality of consumed food, elevated snacking, and worsening of at least one eating behavior were shown [138,139]. Emotional eating due to anxiety and depression have been proposed as the potential causes, as they correlated with diet quality as well as snacking and craving at night-time frequency [138]. Moreover, almost two-thirds of participants limited their physical activity, especially those with a lower BMI loss after surgery or of older age. Furthermore, 45% of the patients complained about increased fatigue, which might be partially associated with shortened sleep duration reported by 30% of the individuals [138].

Several studies aimed directly at the assessment of emotional eating disorders associated with the COVID-19 pandemic have been published so far. In the study by Madali et al., a tremendous number of patients (over 75%) were recognized with eating disorders, likely propelled by the anxiety of contracting SARS-CoV-2 infection and an increased stress level [140]. The prevalence of emotional eating was much higher in obese and overweight individuals than in normal or underweight adults. Almost 80% of the participants reported elevated intake of food [140]. In addition, about one-third affirmed enhanced appetite and weight gain, although no statistically significant increment in BMI values was disclosed [140,141]. Of note, nearly 30% of the individuals declared a more balanced diet, while over 20% admitted unhealthy changes in their nutrition [140]. Interestingly, analogous outcomes were observed in a group of patients with alexithymia characterized by an impaired ability to recognize emotions [142]. Furthermore, over half of the women attending French universities and more than 30% of men were diagnosed with eating disorders [143]. Moreover, Cecchetto et al. revealed a higher prevalence of binge and emotional eating due to pandemic-related emotional distress [144]. Tangentially, psychological distress was suggested as the primary cause of more frequent disordered eating behaviors by Ramalho et al., who showed that 40–50% of adults experienced binge eating episodes or loss of control over eating, while over 80% conceded overeating and grazing eating behavior [141]. Additionally, patients struggling with eating disorders voiced their concerns about being unable to stick with their meal plan and the worsening of eating disorders in general [145]. In the groups of anorexic and bulimic patients, symptoms of eating disorders exacerbated or resurfaced, and isolation, depression, increased anxiety, and media coverage related to weight were the factors associated with the deterioration [146,147]. In addition, a lowering of physical activity was reported during the COVID-19 pandemic among eating-disordered patients, especially those suffering from a hyperphagic eating disorder [140,143]. Furthermore, reduced physical activity elevated the risk of hyperphagic and bulimic eating disorders, creating a vicious circle [143]. On the other hand, compulsive physical activity was reported as a coping strategy, which helped to manage depressive symptoms in anorexic patients, but worsened the underlying disease [147]. Interestingly, sleep duration extension was observed in over 30% of participants reporting emotional eating [140], though this finding was not recapitulated in other research [141].

## 5. Limitations and Biases

Our narrative review has several limitations. First of all, the vast majority of the cited research was based on online surveys. Thus, they were not randomized and were limited to the younger population with high internet skills. Only four articles were characterized by a mean age of over 50 years. Furthermore, several longitudinal papers have been performed until the day of the literature search, and merely a few of them were prospective. Also, most of the studies were conducted during the lockdown periods. In addition, comparing the results from different countries is inherently associated with biases resulting from distinct lockdown policies, dynamics of the COVID-19 pandemic, and cultural diversity. Moreover, the questionnaires used in various studies were not uniform, and many papers present results in their own way, which makes them harder to compare and synthesize. Finally, our review does not include articles published after 1 August 2022, and the PubMed database was the only hefty collection analyzed.

## 6. Conclusions

Unhealthy eating behaviors were a common finding during the COVID-19 pandemic in numerous European countries, albeit differing results were evidenced in a few papers. Increased snacking, intake of caloric foods, such as sweets, pastries, and beverages, and a decline in physical activity were widely reported. Consequently, up to 50% of European adults put on several kilograms. Elevated mental stress and emotional eating were identified as the principal causes of weight gain. Of note, unfavorable alterations in nutrition were more profound in the populations with obesity and overweight. Although only a few follow-up research studies were performed, it seems that unhealthy dietary patterns initiated during COVID-19 lockdowns were mostly maintained over time, while a positive attitude towards immunomodulatory foods and supplements receded. Thus, adverse outcomes of the COVID-19 pandemic may still affect populational health and should constantly attract the attention of public health efforts.

The concluding remarks of our review stay in line with the outcomes of the meta-analysis by Mignogna et al., who also revealed the rise in food intake, snacking, and sweet product use [84]. However, increased consumption of fruits and vegetables was reported much more frequently in the Mediterranean countries than in the rest of Europe, as well as an elevated adherence to the Mediterranean diet and improved overall diet quality [84]. Further meta-analyses, including the weight gain measure and changes in physical activity, not covered by Mignogna et al. [84], need to be performed.

## Figures and Tables

**Table 1:** Immunomodulatory effects of trace elements and vitamins.

	Activity	Immune-Mechanism	Dietary Source
Zinc	- prevents SARS-CoV-2 invasion and directly suppresses viral replication	- zinc-finger protein ZCCHC3 binds RNA and helps to recognize viral RNA in the host cell by activating retinoic acid-inducible gene-I [20]	Meat, liver, rennet cheese, dark bread, buckwheat, and eggs
		- through RIG-1-like receptors, zinc upregulates the interferon type 1 response, which results in the synthesis of antiviral proteins [21]	
		- inhibits Viral RNA-dependent RNA polymerase (RdRp) [22]	
		- upregulates NK cell activity and IL-2 secretion, promoting antiviral immune response [23,24]	
	- limits oxidative stress and normalizes excessive cytokine release	- stimulates the synthesis of glutathione [25]	
		- inhibits pro-inflammatory transcription nuclear factor κB (NF-κB) [26]	
		- inhibits IL-6 production; Zn level correlates positively with IFN-γ [27]	
		- reduces TNF-α, IL-1β, IL-6, MCP-1, vascular cell adhesion molecule (VCAM), and CRP [28]	
	- is required for B and Th cell maturation, number, and function	- facilitates antiapoptotic signaling and cell survival in early B-cell development in the bone marrow [29]	
		- activity of biologically active form of thymulin, which mediates maturation of T helper cells, requires the presence of Zn^2+^ in its molecule [30,31]	
		- regulates CD4+/CD8+ T cell count [32]	
	- reduces oxidative stress, which limits uncontrolled inflammatory response and cytokine release	- maintains the redox balance via superoxide dismutase that contains zinc and copper and affects the expression of glutamate–cysteine ligase [33] - stimulates glutathione biosynthesis [34]	
Iron	- iron overload and high ferritin is associated with iron toxicity	- pro-inflammatory IL-6 stimulates ferritin and the synthesis of hepcidin, which leads to intracellular iron excess, oxidative stress, and ferroptosis [35,36]	Heme iron: giblets, meat, and eggs; nonheme iron: parsley, pulses, and dark bread
	- mild to moderate iron deficiency mitigates viral infection	- SARS-CoV-2 is highly dependent on iron, and iron deficiency might be protective from ferroptosis and viral spread [37,38,39]	
	- iron deficiency negatively affects cell-mediated immunity in children	- iron deficiency in children is related to a reduction in the CD4+ count, decreased CD4:CD8 ratio, and decreased maturation of T cells [40]	
Copper	- neutralizes RNA viruses	- causes RNA damage through the generation of lethal hydroxyl radicals [41] - exposure to copper destroys the viral genome via premature virus uncoating and degradation of the exposed vRNA [42,43] - cold-sprayed copper coatings exhibit a virucidal activity [44]	Wheat bran, oat flakes, offal meat (liver), nuts, cocoa, and sunflower seeds
Selenium	- stimulates cellular and humoral immunity, and innate immune cell functions	- stimulates proliferation of T cells and increases antiviral cytokines like IFN-γ [45]	Offal meat (kidney), shellfish, fish, legume seeds, garlic, and mushrooms
		- increases NK cell cytotoxic effects [45]	
		- increases the expression of IL-2 receptors on B cells [46]	
	- exhibits antioxidant effects and limits inflammation	- maintains the redox balance via selenoproteins that contain selenium, mainly glutathione peroxidases (GPx) and thioredoxin reductases [47] - modulates inflammatory responses, limits pulmonary lipid peroxidation and lung injury in patients with ARDS [48]	
Magnesium	- possibly prevents SARS-CoV-2 invasion	- possibly inhibits transmembrane protease serine protease 2 (TMPRSS2) and the pre-protein convertase furin, the two enzymes involved in the cleavage of S protein and cellular invasion of SARS-CoV-2 [49]	Cereal products, legume seeds, nuts, cocoa, dark chocolate, rennet cheese, fish, potatoes, bananas, and green vegetables
	- affects innate immunity by preventing excessive release of inflammatory mediators and oxidative stress	- stabilizes the membranes of mast cells; limits the production of reactive oxygen species by neutrophils and macrophages [50] - inhibits the production of pro-inflammatory cytokines and stimulates anti-inflammatory cytokines secretion by inducing the conversion of macrophages from M0 to M2 phenotype [51]	
	- regulates adaptive immunity	- is necessary for the expression of the natural killer activating receptor (NKG2D) in NK and CD8+ T cells [52]	
Vitamin A (Retinol)	- plays a role in maintaining first-line defense	- supports the formation and functional maturation of epithelial cells [53]	In the form of beta carotene: carrot, parsley, pulses, spinach, curly kale, broccoli, apricots, peaches, milk, eggs, and butter. In the form of retinol: offal meat (liver), butter, eggs, sea fish, and rennet cheese
	- affects second-line defense (inflammation) of the innate immunity - affects adaptive immunity	- retinoic acid (RA), a metabolite of Vitamin A, decreases the production of IL-12 and TNF-α by macrophages and increases the level of anti-inflammatory IL-10 [54]	
		- RA modulates T cell phenotype and mucosal-homing molecules on lymphocytes [54]	
		- RA modulates the maturation and activity of antigen-presenting cells (APCs) [54]	
		- RA promotes the generation of Treg cells and suppresses Th17 cells [54]	
		- enhances Th2 and inhibits Th1 differentiation [54]	
		- RA promotes the generation of CD8+ effector memory T cells and inhibits central memory CD8+ T cells [54]	
		- depending on type of antigen stimulation, RA enhances IgG and IgA production [54]	
	- AM580, a retinoid derivative, demonstrates direct antiviral activities	- AM580 directly inhibits SREBP-related pathways and replication of coronaviruses in host cells [55,56]	
Vitamin B1 (Thiamine)	- affects innate immunity as an antioxidant and anti-inflammatory vitamin	- limits mast cell degranulation [57]	Pork, meat preparations, legume seeds, wholegrain products, and nuts
		- in macrophages and microglial cells, it down-regulates the expression of inflammatory prostaglandin E2, thromboxane 2, prostacyclin, leukotrienes, NF-κB, iNOS, COX-2, IL-1, IL-6, TNFα, and increases anti-inflammatory IL-10 [57,58]	
	- affects adaptive immunity	- as a cofactor for the tricarboxylic acid cycle (TAC), thiamine controls the activity and number of naive B cells in the Peyer’s patches [59]	
Vitamin B2 (Riboflavin)	- affects innate immunity as an antioxidant and anti-inflammatory vitamin	- in macrophages, it decreases the formation of pro-inflammatory cytokines IL-1 and TNF-α, high-mobility group box 1 (HMGB1) protein, iNOS, NO, heat shock protein 72, and monocyte chemoattractant protein-1 (MCP-1), and increases the expression of anti-inflammatory IL-10 [57]	Milk, rennet cheese, cottage cheese, eggs, and offal meat
	- inhibits viral replication	- together with UV radiation, damages viral nucleoid acids in MERS-CoV (Middle East Respiratory Syndrom Coronavirus) and SARS-CoV-2 [60,61]	
Vitamin B3 (Niacin, vitamin PP)	- affects innate immunity as an anti-inflammatory and antiviral vitamin	- activates nicotinic acid receptor (GPR10) that is present in immune cells (e.g., macrophages), leading to decreased levels of pro-inflammatory cytokines (IL-6, TNF-α), MCP-1, and NF-κB and reduced monocyte chemotaxis [57] - regulates signaling pathways, including the NF-κB, p53, HIF-1 and IL-17, and Th17 cell differentiation [62]	Liver, meat, meat preparations, fish, wholegrain products, and peanuts
Vitamin B5 (Pantothenic acid)	- affects innate and adaptive immunity	- stabilizes the membranes of mast cells and controls the number of macrophages [63]	Food of animal origin, wholegrain products, dry legume seeds, and milk
		- promotes the generation of INFγ and IL-17 by CD4+ [63]	
		- inhibits the production of pro-inflammatory IL-6 and TNF-α in acute lung injury [64]	
	- exhibits paradoxical effect on cytokines	- inhibits neutrophils in producing anti-inflammatory cytokines in early bacterial infection and stimulates them in late infection [63]	
Vitamin B6 (Pyridoxine)	- limits cytokine storm and inflammation	- upregulates IL-10 production that inhibits cytokine release by macrophages [65]	Fish, meat (poultry and pork), offal meat (liver), seeds, nuts, brown rice, bananas, dried apricots, potatoes, red pepper, and tomato juice
	- affects B and T cell function	- deficiency of B6 reduces antibody production, IL-2 level, inhibits T cell proliferation, and increases IL-4 levels [57]	
Vitamin B9 (Folic acid, folate)	- prevents SARS-CoV-2 invasion	- inhibits furin activity and prevents sequence-specific cleavage of the spike protein into S1 and S2 domains [66] - inhibits S1-glycoprotein/NRP-1 complex formation [67]	Legume seeds, kale, spinach, lettuce, eggs, offal meat (liver), and rennet cheese
	- normalizes excessive cytokine release - affects the number and function of T cells - affects humoral immunity	- inhibits pro-inflammatory transcription nuclear factor κB (NF-κB) [68]	
		- folate deficiency reduces T cell proliferation and increases the CD4+/CD8+ ratio [68]	
		- is necessary for the survival and differentiation of Treg cells [68]	
		- deficiency of folic acid indirectly affects the proper formation of antibodies [68]	
Vitamin B12 (Cobalamin)	- prevents SARS-CoV-2 invasion and replication	- inhibits furin activity [69] - possibly inhibits viral replication by suppression of the RNA-dependent-RNA polymerase activity of the SCV2-nsp12 enzyme [70]	Meat, fish, eggs, offal meat, and shellfish
	- regulates cytokine release - affects innate immunity - affects humoral immunity	- deficiency of B12 upregulates the synthesis of TNF-α by macrophages and downregulates IL-6 levels [68]	
		- supports NK-cell activity [71]	
		- supports antibody production [71]	
	- affects T cell count and function	- increases CD8+ cell count, reduces CD4+/CD8+ ratio, and stimulates NK cell activity [72]	
Vitamin C (Ascorbic acid)	- affects innate and adaptive immunity	- potentiates differentiation and proliferation of phagocytes and B and T cells [73]	Parsley, black currant, kiwi, red pepper, cabbage vegetables, strawberries, and citrus fruits
		- supports antibody production [73]	
		- supports the development of NK cells and stimulates their activity [73,74]	
		- increases the production of IFN-α/β in viral infections [73]	
		- inhibits pro-inflammatory NFκB pathway and modulates cytokine production [73]	
		- reduces IFN-γ, TNF-α, and IL-6, and increases anti-inflammatory IL-10 production [73]	
	- limits oxidative stress	- scavenges reactive oxygen species (superoxide and peroxyl radicals, hydrogen peroxide, and hypochlorous acid) [73]	
Vitamin D	- plays a role in maintaining first-line defense	- upregulates genes that encode proteins maintaining membrane integrity [75]	Only 20% of vitamin D in the organism comes from the diet, mainly from fatty fish and eggs
		- stimulates maturation of monocytes and phagocytosis [75]	
	- affects second-line defense (inflammation) of the innate immunity and limits cytokine storm - affects B and T cells and limits inflammation	- inhibits monocytes to produce pro-inflammatory IL-1α, IL-1β, IL-6, IL-8, IL-12, and TNFα [76]	
		- suppresses proliferation of B cells and antibody production [77]	
		- inhibits proliferation of T cells and supports a shift from Th1 to Th2 development, which results in reduced pro-inflammatory cytokines production (IFN-γ, TNF-α, IL-2, and IL-12), and increased formation of anti-inflammatory cytokines (IL-10, IL-4, and IL-5) [77]	
		- inhibits Th17 cells and formation of IL-6, IL-17, and IL-23 [77,78]	
		- induces Treg cells [77]	
Vitamin E (Tocopherol)	- directly suppresses SARS-CoV-2 replication	- water-soluble derivatives of α-tocopherol inhibit SARS-CoV-2 RNA-dependent RNA polymerase [79]	Vegetable oils, grain products, nuts, vegetables, meat products, and dairy products
	- affects effector cells of innate immunity	- inhibits COX2 activity and PGE2 production by macrophages [80,81]	
		- increases the cytotoxicity of NK cells, possibly by modulating NO levels [80,81]	
		- inhibits klotho sensitive NF-κB-mediated dendritic cell maturation, migration, and function [82]	
		- stimulates proliferation of naïve T cells and their secretion of IL-2 [82]	
	- affects adaptive immunity	- enhances Th1 response [82]	
	- limits oxidative stress and normalizes excessive cytokine release	- decreases the production of reactive oxygen species by monocytes and dendritic cells [83]	
		- downregulates IL1, IL-6, and TNF secretion by monocytes and macrophages [81]	
		- inhibits pro-inflammatory eicosanoids (PGE2, LTB4, and 8-isoprostane) formation [81]	

**Table 2:** Summary of the included studies.

Ref.	Type of Study	Dates of the Study	Pandemic Restrictions	N	Mean Age ± SD [Years]	Country	Type of Survey
[85]	cross-sectional	17.04–01.05.2020	lockdown	1097	27.7 ± 9.0	Poland	online
[86]	cross-sectional	03–04.2020	lockdown	183	33 ± 11	Poland	online
[87]	longitudinal retrospective	29.04–19.05.2020	pre-lockdown and lockdown	312	42.1 ± 12.0 M 40.6 ± 13.7 F	Poland	online
[88]	cross-sectional	30.04–23.05.2020	lockdown	2381	not reported	Poland	online
[89]	cross-sectional	14.04–28.04.2020	lockdown	2447	not reported	Lithuania	online
[90]	cross-sectional	03.05–06.06.2021	post-lockdown	2040	not reported	Romania	online
[91]	cross-sectional	29.04–19.05.2020	lockdown	312	41.12 ± 13.05	Poland	online
[92]	longitudinal prospective	03.2020 and 10.2020	lockdown and post-lockdown	200	19.5 ± 0.6	Poland	online
[93]	longitudinal retrospective	24.03–11.04.2020	pre-lockdown and lockdown	506	24.67 ± 4.23	Poland	online
[94]	cross-sectional	10–11.02.2021	post-lockdown	108	46.3 ± 10.5	Poland	paper
[95]	longitudinal retrospective	01.06–01.08.2020	post-lockdown	171	22.53 ± 2.48	Serbia	online and paper
[96]	cross-sectional	05.05–30.06.2020	lockdown	689	not reported	Kosovo	online
[97]	cross-sectional	10.2019–06.2020 and 11.2020–03.2021	pre-lockdown and post-lockdown	6369 and 2392	37.9 ± 11.8 and 38.4 ± 12.6	Lithuania	online
[98]	cross-sectional	20.03–30.05.2020	lockdown	926	not reported	Poland	online
[99]	cross-sectional	04–05.2020	lockdown	1082	31.6 ± 11.98	Poland	online
[100]	cross-sectional	01.01–20.06.2021	post-lockdown	1022	33.18 ± 11.86	Poland	online
[101]	cross-sectional	01.05–15.05.2021	post-lockdown	145	not reported	Poland	online
[102]	cross-sectional	04–12.2020	lockdown	9936	31.0 ± 12.16	Russia	online
[103]	cross-sectional	04–05.2020 and 11.2020	lockdown and post-lockdown	978	not reported	Poland	online
[104]	cross-sectional	01.11.2020–31.01.2021	post-lockdown	1101	not reported	Poland	online
[105]	cross-sectional	01–03.2021	post-lockdown	1447	31.34 ± 11.05	Poland	online
[106]	cross-sectional	22.02–03.04.2021	post-lockdown	1323	22 ± 4	Poland	online
[107]	cross-sectional	20.03–15.12.2021	post-lockdown	894	20.73 ± 1.81	Poland	paper
[108]	longitudinal retrospective	11.2020–01.2021	pre- and post-lockdown	435	not reported	Poland	online
[109]	cohort	08.2019–03.2020, 03–06.2020, and 06–08.2020	pre-lockdown, lockdown, and post-lockdown	1877	26.5 ± 6.8	Sweden	online
[110]	cross-sectional	24.04–05.05.2020	lockdown	2462	not reported	Denmark	online
[111]	cross-sectional	15–30.04.2020	lockdown	24,968	not reported	Norway	online
[112]	cross-sectional	03–04.2020	lockdown	1964	23.3 ± 4.0	Germany	online
[113]	cross-sectional	12.03–03.05.2020	lockdown	827	not reported	Germany	online
[114]	cross-sectional	12.04–03.05.2020	lockdown	2103	40 ± 14	Germany	online
[115]	longitudinal retrospective	11.2020	lockdown and post-lockdown	1694	47.6 ±14.8	France	online
[116]	cross-sectional	25–30.03.2020	lockdown	11,391	47.47 ± 17.28	France	online
[117]	longitudinal retrospective	30.04–01.05.2020	pre-lockdown and lockdown	938	38.7 ± 11.6	France	online
[118]	cross-sectional	09–30.06.2020	lockdown	2422	not reported	France	online
[119]	cross-sectional	2009, 2012, 2015, 2018, and 05.2021	pre- and post-lockdown	8981	20.4 ± 1.8	France	online
[120]	cross-sectional	22–28.04.2020	lockdown	1030	49.9 ± 17.0	Netherlands	online
[121]	cross-sectional	9–22.04.2020	lockdown	1129	34.9 ± 14.3	Belgium	online
[122]	cross-sectional	16–23.04.2020	lockdown	28,029	not reported	Belgium	online
[123]	cross-sectional	08.06–08.10.2020	lockdown and post-lockdown	1119	74 ± 7	Netherlands	paper, online, and phone
[124]	cross-sectional	03–05.2020	lockdown	8122	not reported	Belgium	online
[125]	longitudinal prospective	01.07–18.11.2020	post-lockdown	246	49.6 ± 17.2	Netherlands	online
[126]	longitudinal retrospective	29.04–13.05.2020	lockdown	818	47 ± 13	England	online
[127]	cross-sectional	11–12.2020	lockdown	792	not reported	England	online
[128]	cross-sectional	15.05–27.06.2020	lockdown	588	33.4 ± 12.6	United Kingdom	online
[129]	cross-sectional	28.04–22.05.2020	lockdown	2002	34.74 ± 12.3	United Kingdom	online
[130]	cross-sectional	01–15.09.2020	post-lockdown	352	not reported	United Kingdom	online
[131]	longitudinal prospective	10.2019 and 02.2021	pre-lockdown and lockdown	230	23.9 ± 5.4	Lithuania	online
[132]	longitudinal prospective	28.03–29.05.2020	lockdown	22,374	not reported	United Kingdom	online
[133]	cross-sectional	16.03–30.11.2020	lockdown and post-lockdown	37,988	57.36 ± 8.23	United Kingdom	not reported
[134]	cross-sectional	03–05.2020	lockdown	442	45 ± 12.7	Turkey	online
[135]	longitudinal retrospective	06–22.07.2020	post-lockdown	124	23	Poland	online
[136]	cross-sectional	08.04–20.05.2020	lockdown	72	63	Spain	phone
[137]	cross-sectional	04–07.05.2020	lockdown	284	60.4 ± 10.8	Spain	phone
[138]	cross-sectional	27.04–27.05.2020 and 07–15.12.2020	lockdown	420	50.3 ± 12.0	France	phone and online
[139]	longitudinal prospective	not reported	pre-lockdown and lockdown	66 35	50.06 ± 10.68 50.80 ± 12.40	Portugal	phone and online
[140]	cross-sectional	08–09.2020	lockdown	1626	30 ± 11	Turkey	online
[141]	cross-sectional	11–25.05.2020	post-lockdown	254	35.82 ± 11.82	Portugal	online
[142]	cross-sectional	07.2020	post-lockdown	158	32 ± 11.88	United Kingdom	online
[143]	cross-sectional	05.2021	post-lockdown	3058	20.7 ± 2.3	France	online
[144]	longitudinal retrospective	14–19.05.2020	lockdown and post-lockdown	365	35.09 ± 13.59	Italy	online
[145]	cross-sectional	17.04–15.05.2020	lockdown	511	not reported	Netherlands	online
[146]	longitudinal prospective	01–09.2019 11.2019–01.2020, 22.04–03.05.2020	pre-lockdown and lockdown	171	31.74 ± 12.76	Italy	online, phone, and direct
[147]	cross-sectional	07.05–12.06.2020	lockdown	32	35.2 ± 10.3	United Kingdom	online

F—females; M—males; Ref.—reference; and SD—standard deviation.

## Data Availability

Not applicable.

## References

[1] Talic S., Shah S., Wild H., Gasevic D., Maharaj A., Ademi Z., Li X., Xu W., Mesa-Eguiagaray I., Rostron J. (2021). Effectiveness of Public Health Measures in Reducing the Incidence of COVID-19, SARS-CoV-2 Transmission, and COVID-19 Mortality: Systematic Review and Meta-Analysis. BMJ.

[2] Polack F.P., Thomas S.J., Kitchin N., Absalon J., Gurtman A., Lockhart S., Perez J.L., Pérez Marc G., Moreira E.D., Zerbini C. (2020). Safety and Efficacy of the BNT162b2 MRNA COVID-19 Vaccine. N. Engl. J. Med..

[3] Baden L.R., El Sahly H.M., Essink B., Kotloff K., Frey S., Novak R., Diemert D., Spector S.A., Rouphael N., Creech C.B. (2021). Efficacy and Safety of the MRNA-1273 SARS-CoV-2 Vaccine. N. Engl. J. Med..

[4] Sadoff J., Gray G., Vandebosch A., Cárdenas V., Shukarev G., Grinsztejn B., Goepfert P.A., Truyers C., Fennema H., Spiessens B. (2021). Safety and Efficacy of Single-Dose Ad26.COV2.S Vaccine against COVID-19. N. Engl. J. Med..

[5] Voysey M., Clemens S.A.C., Madhi S.A., Weckx L.Y., Folegatti P.M., Aley P.K., Angus B., Baillie V.L., Barnabas S.L., Bhorat Q.E. (2021). Safety and Efficacy of the ChAdOx1 NCoV-19 Vaccine (AZD1222) against SARS-CoV-2: An Interim Analysis of Four Randomised Controlled Trials in Brazil, South Africa, and the UK. Lancet.

[6] Heath P.T., Galiza E.P., Baxter D.N., Boffito M., Browne D., Burns F., Chadwick D.R., Clark R., Cosgrove C., Galloway J. (2021). Safety and Efficacy of NVX-CoV2373 COVID-19 Vaccine. N. Engl. J. Med..

[7] Tanriover M.D., Doğanay H.L., Akova M., Güner H.R., Azap A., Akhan S., Köse Ş., Erdinç F.Ş., Akalın E.H., Tabak Ö.F. (2021). Efficacy and Safety of an Inactivated Whole-Virion SARS-CoV-2 Vaccine (CoronaVac): Interim Results of a Double-Blind, Randomised, Placebo-Controlled, Phase 3 Trial in Turkey. Lancet.

[8] Drożdżal S., Rosik J., Lechowicz K., Machaj F., Szostak B., Przybyciński J., Lorzadeh S., Kotfis K., Ghavami S., Łos M.J. (2021). An Update on Drugs with Therapeutic Potential for SARS-CoV-2 (COVID-19) Treatment. Drug Resist. Updat..

[9] COVID-19 Map. https://coronavirus.jhu.edu/map.html.

[10] Górska P., Górna I., Miechowicz I., Przysławski J. (2021). Changes in Life Situations during the SARS-CoV-2 Virus Pandemic and Their Impact on Eating Behaviors for Residents of Europe, Australia as Well as North and South America. Nutrients.

[11] Dizdar O.S., Baspınar O., Kocer D., Dursun Z.B., Avcı D., Karakükcü C., Çelik İ., Gundogan K. (2016). Nutritional Risk, Micronutrient Status and Clinical Outcomes: A Prospective Observational Study in an Infectious Disease Clinic. Nutrients.

[12] Jagielski P., Łuszczki E., Wnęk D., Micek A., Bolesławska I., Piórecka B., Kawalec P. (2022). Associations of Nutritional Behavior and Gut Microbiota with the Risk of COVID-19 in Healthy Young Adults in Poland. Nutrients.

[13] Iddir M., Brito A., Dingeo G., Fernandez Del Campo S.S., Samouda H., La Frano M.R., Bohn T. (2020). Strengthening the Immune System and Reducing Inflammation and Oxidative Stress through Diet and Nutrition: Considerations during the COVID-19 Crisis. Nutrients.

[14] Chattopadhyay I., Shankar E.M. (2021). SARS-CoV-2-Indigenous Microbiota Nexus: Does Gut Microbiota Contribute to Inflammation and Disease Severity in COVID-19?. Front. Cell Infect. Microbiol..

[15] de Oliveira G.L.V., Oliveira C.N.S., Pinzan C.F., de Salis L.V.V., Cardoso C.R.D.B. (2021). Microbiota Modulation of the Gut-Lung Axis in COVID-19. Front. Immunol..

[16] Muscogiuri G., Barrea L., Savastano S., Colao A. (2020). Nutritional Recommendations for COVID-19 Quarantine. Eur. J. Clin. Nutr..

[17] de Faria Coelho-Ravagnani C., Corgosinho F.C., Sanches F.L.F.Z., Prado C.M.M., Laviano A., Mota J.F. (2021). Dietary Recommendations during the COVID-19 Pandemic. Nutr. Rev..

[18] Che Abdul Rahim N., Manjit Singh J.S., Pardi M., Zainuddin A.A., Salleh R. (2021). Analysis of Available Nutrition Recommendations to Combat COVID-19: A Scoping Review. Malays. J. Med. Sci..

[19] Detopoulou P., Tsouma C., Papamikos V. (2022). COVID-19 and Nutrition: Summary of Official Recommendations. Top. Clin. Nutr..

[20] Lian H., Zang R., Wei J., Ye W., Hu M.-M., Chen Y.-D., Zhang X.-N., Guo Y., Lei C.-Q., Yang Q. (2018). The Zinc-Finger Protein ZCCHC3 Binds RNA and Facilitates Viral RNA Sensing and Activation of the RIG-I-like Receptors. Immunity.

[21] Berg K., Bolt G., Andersen H., Owen T.C. (2001). Zinc Potentiates the Antiviral Action of Human IFN-Alpha Tenfold. J. Interferon Cytokine Res..

[22] te Velthuis A.J.W., van den Worm S.H.E., Sims A.C., Baric R.S., Snijder E.J., van Hemert M.J. (2010). Zn^2+^ Inhibits Coronavirus and Arterivirus RNA Polymerase Activity in Vitro and Zinc Ionophores Block the Replication of These Viruses in Cell Culture. PLoS Pathog..

[23] Kloubert V., Wessels I., Wolf J., Blaabjerg K., Janssens V., Hapala J., Wagner W., Rink L. (2021). Zinc Deficiency Leads to Reduced Interleukin-2 Production by Active Gene Silencing Due to Enhanced CREMα Expression in T Cells. Clin. Nutr..

[24] Ravaglia G., Forti P., Maioli F., Bastagli L., Facchini A., Mariani E., Savarino L., Sassi S., Cucinotta D., Lenaz G. (2000). Effect of Micronutrient Status on Natural Killer Cell Immune Function in Healthy Free-Living Subjects Aged >/=90 y. Am. J. Clin. Nutr..

[25] Eide D.J. (2011). The Oxidative Stress of Zinc Deficiency. Metallomics.

[26] Prasad A.S., Bao B., Beck F.W.J., Sarkar F.H. (2011). Zinc-Suppressed Inflammatory Cytokines by Induction of A20-Mediated Inhibition of Nuclear Factor-ΚB. Nutrition.

[27] Prasad A.S., Beck F.W.J., Bao B., Fitzgerald J.T., Snell D.C., Steinberg J.D., Cardozo L.J. (2007). Zinc Supplementation Decreases Incidence of Infections in the Elderly: Effect of Zinc on Generation of Cytokines and Oxidative Stress. Am. J. Clin. Nutr..

[28] Bao B., Prasad A.S., Beck F.W.J., Godmere M. (2003). Zinc Modulates MRNA Levels of Cytokines. Am. J. Physiol. Endocrinol. Metab..

[29] Miyai T., Hojyo S., Ikawa T., Kawamura M., Irié T., Ogura H., Hijikata A., Bin B.-H., Yasuda T., Kitamura H. (2014). Zinc Transporter SLC39A10/ZIP10 Facilitates Antiapoptotic Signaling during Early B-Cell Development. Proc. Natl. Acad. Sci. USA.

[30] Dardenne M., Savino W., Wade S., Kaiserlian D., Lemonnier D., Bach J.F. (1984). In Vivo and In Vitro Studies of Thymulin in Marginally Zinc-Deficient Mice. Eur. J. Immunol..

[31] Hojyo S., Fukada T. (2016). Roles of Zinc Signaling in the Immune System. J. Immunol. Res..

[32] Prasad A.S. (2000). Effects of Zinc Deficiency on Th1 and Th2 Cytokine Shifts. J. Infect. Dis..

[33] Marreiro D.D.N., Cruz K.J.C., Morais J.B.S., Beserra J.B., Severo J.S., de Oliveira A.R.S. (2017). Zinc and Oxidative Stress: Current Mechanisms. Antioxidants.

[34] Cortese M.M., Suschek C.V., Wetzel W., Kröncke K.-D., Kolb-Bachofen V. (2008). Zinc Protects Endothelial Cells from Hydrogen Peroxide via Nrf2-Dependent Stimulation of Glutathione Biosynthesis. Free Radic. Biol. Med..

[35] Edeas M., Saleh J., Peyssonnaux C. (2020). Iron: Innocent Bystander or Vicious Culprit in COVID-19 Pathogenesis?. Int. J. Infect. Dis..

[36] Wojciechowska M., Wisniewski O.W., Kolodziejski P., Krauss H. (2021). Role of Hepcidin in Physiology and Pathophysiology. Emerging Experimental and Clinical Evidence. J. Physiol. Pharmacol..

[37] Menshawey R., Menshawey E., Alserr A.H.K., Abdelmassih A.F. (2020). Low Iron Mitigates Viral Survival: Insights from Evolution, Genetics, and Pandemics—A Review of Current Hypothesis. Egypt. J. Med. Hum. Genet..

[38] Zhao K., Huang J., Dai D., Feng Y., Liu L., Nie S. (2020). Serum Iron Level as a Potential Predictor of Coronavirus Disease 2019 Severity and Mortality: A Retrospective Study. Open Forum Infect. Dis..

[39] Szabo R., Petrisor C., Bodolea C., Simon R., Maries I., Tranca S., Mocan T. (2022). Hyperferritinemia, Low Circulating Iron and Elevated Hepcidin May Negatively Impact Outcome in COVID-19 Patients: A Pilot Study. Antioxidants.

[40] Aly S.S., Fayed H.M., Ismail A.M., Abdel Hakeem G.L. (2018). Assessment of Peripheral Blood Lymphocyte Subsets in Children with Iron Deficiency Anemia. BMC Pediatr..

[41] Borkow G., Gabbay J. (2005). Copper as a Biocidal Tool. Curr. Med. Chem..

[42] Warnes S.L., Little Z.R., Keevil C.W. (2015). Human Coronavirus 229E Remains Infectious on Common Touch Surface Materials. mBio.

[43] Monette A., Mouland A.J. (2020). Zinc and Copper Ions Differentially Regulate Prion-Like Phase Separation Dynamics of Pan-Virus Nucleocapsid Biomolecular Condensates. Viruses.

[44] Hutasoit N., Kennedy B., Hamilton S., Luttick A., Rahman Rashid R.A., Palanisamy S. (2020). SARS-CoV-2 (COVID-19) Inactivation Capability of Copper-Coated Touch Surface Fabricated by Cold-Spray Technology. Manuf. Lett..

[45] Huang Z., Rose A.H., Hoffmann P.R. (2012). The Role of Selenium in Inflammation and Immunity: From Molecular Mechanisms to Therapeutic Opportunities. Antioxid. Redox Signal..

[46] Roy M., Kiremidjian-Schumacher L., Wishe H.I., Cohen M.W., Stotzky G. (1993). Selenium Supplementation Enhances the Expression of Interleukin 2 Receptor Subunits and Internalization of Interleukin 2. Proc. Soc. Exp. Biol. Med..

[47] Arthur J.R., McKenzie R.C., Beckett G.J. (2003). Selenium in the Immune System. J. Nutr..

[48] Mahmoodpoor A., Hamishehkar H., Shadvar K., Ostadi Z., Sanaie S., Saghaleini S.H., Nader N.D. (2019). The Effect of Intravenous Selenium on Oxidative Stress in Critically Ill Patients with Acute Respiratory Distress Syndrome. Immunol. Investig..

[49] Trapani V., Rosanoff A., Baniasadi S., Barbagallo M., Castiglioni S., Guerrero-Romero F., Iotti S., Mazur A., Micke O., Pourdowlat G. (2022). The Relevance of Magnesium Homeostasis in COVID-19. Eur. J. Nutr..

[50] Maier J.A., Castiglioni S., Locatelli L., Zocchi M., Mazur A. (2021). Magnesium and Inflammation: Advances and Perspectives. Semin. Cell Dev. Biol..

[51] Sun L., Li X., Xu M., Yang F., Wang W., Niu X. (2020). In Vitro Immunomodulation of Magnesium on Monocytic Cell toward Anti-Inflammatory Macrophages. Regen. Biomater..

[52] Chaigne-Delalande B., Li F.-Y., O’Connor G.M., Lukacs M.J., Jiang P., Zheng L., Shatzer A., Biancalana M., Pittaluga S., Matthews H.F. (2013). Mg^2+^ Regulates Cytotoxic Functions of NK and CD8 T Cells in Chronic EBV Infection through NKG2D. Science.

[53] McCullough F.S., Northrop-Clewes C.A., Thurnham D.I. (1999). The Effect of Vitamin A on Epithelial Integrity. Proc. Nutr. Soc..

[54] Larange A., Cheroutre H. (2016). Retinoic Acid and Retinoic Acid Receptors as Pleiotropic Modulators of the Immune System. Annu. Rev. Immunol..

[55] Yuan S., Chu H., Chan J.F.-W., Ye Z.-W., Wen L., Yan B., Lai P.-M., Tee K.-M., Huang J., Chen D. (2019). SREBP-Dependent Lipidomic Reprogramming as a Broad-Spectrum Antiviral Target. Nat. Commun..

[56] Yuan S., Chan C.C.-Y., Chik K.K.-H., Tsang J.O.-L., Liang R., Cao J., Tang K., Cai J.-P., Ye Z.-W., Yin F. (2020). Broad-Spectrum Host-Based Antivirals Targeting the Interferon and Lipogenesis Pathways as Potential Treatment Options for the Pandemic Coronavirus Disease 2019 (COVID-19). Viruses.

[57] Mikkelsen K., Apostolopoulos V., Mahmoudi M., Rezaei N. (2019). Vitamin B1, B2, B3, B5, and B6 and the Immune System. Nutrition and Immunity.

[58] Menezes R.R., Godin A.M., Rodrigues F.F., Coura G.M.E., Melo I.S.F., Brito A.M.S., Bertollo C.M., Paulino T.P., Rachid M.A., Machado R.R. (2017). Thiamine and Riboflavin Inhibit Production of Cytokines and Increase the Anti-Inflammatory Activity of a Corticosteroid in a Chronic Model of Inflammation Induced by Complete Freund’s Adjuvant. Pharmacol. Rep..

[59] Kunisawa J., Sugiura Y., Wake T., Nagatake T., Suzuki H., Nagasawa R., Shikata S., Honda K., Hashimoto E., Suzuki Y. (2015). Mode of Bioenergetic Metabolism during B Cell Differentiation in the Intestine Determines the Distinct Requirement for Vitamin B1. Cell Rep..

[60] Ragan I., Hartson L., Pidcoke H., Bowen R., Goodrich R. (2020). Pathogen Reduction of SARS-CoV-2 Virus in Plasma and Whole Blood Using Riboflavin and UV Light. PLoS ONE.

[61] Keil S.D., Ragan I., Yonemura S., Hartson L., Dart N.K., Bowen R. (2020). Inactivation of Severe Acute Respiratory Syndrome Coronavirus 2 in Plasma and Platelet Products Using a Riboflavin and Ultraviolet Light-Based Photochemical Treatment. Vox Sang..

[62] Li R., Li Y., Liang X., Yang L., Su M., Lai K.P. (2021). Network Pharmacology and Bioinformatics Analyses Identify Intersection Genes of Niacin and COVID-19 as Potential Therapeutic Targets. Brief. Bioinform..

[63] Gheita A.A., Gheita T.A., Kenawy S.A. (2020). The Potential Role of B5: A Stitch in Time and Switch in Cytokine. Phytother. Res..

[64] Li-Mei W., Jie T., Shan-He W., Dong-Mei M., Peng-Jiu Y. (2016). Anti-Inflammatory and Anti-Oxidative Effects of Dexpanthenol on Lipopolysaccharide Induced Acute Lung Injury in Mice. Inflammation.

[65] Mikkelsen K., Prakash M.D., Kuol N., Nurgali K., Stojanovska L., Apostolopoulos V. (2019). Anti-Tumor Effects of Vitamin B2, B6 and B9 in Promonocytic Lymphoma Cells. Int. J. Mol. Sci..

[66] Sheybani Z., Heydari Dokoohaki M., Negahdaripour M., Dehdashti M., Zolghadr H., Moghadami M., Masoompour S.M., Zolghadr A.R. (2021). The Interactions of Folate with the Enzyme Furin: A Computational Study. RSC Adv..

[67] Škrbić R., Travar M., Stojiljković M.P., Djuric D.M., Suručić R. (2023). Folic Acid and Leucovorin Have Potential to Prevent SARS-CoV-2-Virus Internalization by Interacting with S-Glycoprotein/Neuropilin-1 Receptor Complex. Molecules.

[68] Mikkelsen K., Apostolopoulos V., Mahmoudi M., Rezaei N. (2019). Vitamin B12, Folic Acid, and the Immune System. Nutrition and Immunity.

[69] Pandya M., Shah S., Dhanalakshmi M., Juneja T., Patel A., Gadnayak A., Dave S., Das K., Das J. (2022). Unravelling Vitamin B12 as a Potential Inhibitor against SARS-CoV-2: A Computational Approach. Inform. Med. Unlocked.

[70] Narayanan N., Nair D.T. (2020). Vitamin B12 May Inhibit RNA-Dependent-RNA Polymerase Activity of Nsp12 from the SARS-CoV-2 Virus. IUBMB Life.

[71] Calder P.C. (2021). Nutrition and Immunity: Lessons for COVID-19. Eur. J. Clin. Nutr..

[72] Tamura J., Kubota K., Murakami H., Sawamura M., Matsushima T., Tamura T., Saitoh T., Kurabayshi H., Naruse T. (1999). Immunomodulation by Vitamin B12: Augmentation of CD8+ T Lymphocytes and Natural Killer (NK) Cell Activity in Vitamin B12-Deficient Patients by Methyl-B12 Treatment. Clin. Exp. Immunol..

[73] Farjana M., Moni A., Sohag A.A.M., Hasan A., Hannan M.A., Hossain M.G., Uddin M.J. (2020). Repositioning Vitamin C as a Promising Option to Alleviate Complications Associated with COVID-19. Infect. Chemother..

[74] Kim Y., Kim H., Bae S., Choi J., Lim S.Y., Lee N., Kong J.M., Hwang Y.-I., Kang J.S., Lee W.J. (2013). Vitamin C Is an Essential Factor on the Anti-Viral Immune Responses through the Production of Interferon-α/β at the Initial Stage of Influenza A Virus (H3N2) Infection. Immune Netw..

[75] Rondanelli M., Miccono A., Lamburghini S., Avanzato I., Riva A., Allegrini P., Faliva M.A., Peroni G., Nichetti M., Perna S. (2018). Self-Care for Common Colds: The Pivotal Role of Vitamin D, Vitamin C, Zinc, and Echinacea in Three Main Immune Interactive Clusters (Physical Barriers, Innate and Adaptive Immunity) Involved during an Episode of Common Colds-Practical Advice on Dosages and on the Time to Take These Nutrients/Botanicals in Order to Prevent or Treat Common Colds. Evid.-Based Complement. Altern. Med..

[76] Zhang Y., Leung D.Y.M., Richers B.N., Liu Y., Remigio L.K., Riches D.W., Goleva E. (2012). Vitamin D Inhibits Monocyte/Macrophage Proinflammatory Cytokine Production by Targeting MAPK Phosphatase-1. J. Immunol..

[77] Aranow C. (2011). Vitamin D and the Immune System. J. Investig. Med..

[78] Daniel C., Sartory N.A., Zahn N., Radeke H.H., Stein J.M. (2008). Immune Modulatory Treatment of Trinitrobenzene Sulfonic Acid Colitis with Calcitriol Is Associated with a Change of a T Helper (Th) 1/Th17 to a Th2 and Regulatory T Cell Profile. J. Pharmacol. Exp. Ther..

[79] Pacl H.T., Tipper J.L., Sevalkar R.R., Crouse A., Crowder C., Ahmad S., Ahmad A., Holder G.D., Kuhlman C.J., UAB Precision Medicine Institute (2021). Water-Soluble Tocopherol Derivatives Inhibit SARS-CoV-2 RNA-Dependent RNA Polymerase. bioRxiv.

[80] O’Leary K.A., de Pascual-Teresa S., Needs P.W., Bao Y.-P., O’Brien N.M., Williamson G. (2004). Effect of Flavonoids and Vitamin E on Cyclooxygenase-2 (COX-2) Transcription. Mutat. Res..

[81] Jiang Q. (2014). Natural Forms of Vitamin E: Metabolism, Antioxidant, and Anti-Inflammatory Activities and Their Role in Disease Prevention and Therapy. Free Radic. Biol. Med..

[82] Xuan N.T., Trang P.T.T., Van Phong N., Toan N.L., Trung D.M., Bac N.D., Nguyen V.L., Hoang N.H., Van Hai N. (2016). Klotho Sensitive Regulation of Dendritic Cell Functions by Vitamin E. Biol. Res..

[83] Singh U., Devaraj S., Jialal I. (2005). Vitamin E, Oxidative Stress, and Inflammation. Annu. Rev. Nutr..

[84] Mignogna C., Costanzo S., Ghulam A., Cerletti C., Donati M.B., de Gaetano G., Iacoviello L., Bonaccio M. (2021). Impact of Nationwide Lockdowns Resulting from The First Wave of the COVID-19 Pandemic on Food Intake, Eating Behaviours and Diet Quality: A Systematic Review. Adv. Nutr..

[85] Sidor A., Rzymski P. (2020). Dietary Choices and Habits during COVID-19 Lockdown: Experience from Poland. Nutrients.

[86] Dobrowolski H., Włodarek D. (2021). Body Mass, Physical Activity and Eating Habits Changes during the First COVID-19 Pandemic Lockdown in Poland. Int. J. Environ. Res. Public Health.

[87] Bolesławska I., Błaszczyk-Bębenek E., Jagielski P., Jagielska A., Przysławski J. (2021). Nutritional Behaviors of Women and Men in Poland during Confinement Related to the SARS-CoV-2 Epidemic. Sci. Rep..

[88] Górnicka M., Drywień M.E., Zielinska M.A., Hamułka J. (2020). Dietary and Lifestyle Changes During COVID-19 and the Subsequent Lockdowns among Polish Adults: A Cross-Sectional Online Survey PLife COVID-19 Study. Nutrients.

[89] Kriaucioniene V., Bagdonaviciene L., Rodríguez-Pérez C., Petkeviciene J. (2020). Associations between Changes in Health Behaviours and Body Weight during the COVID-19 Quarantine in Lithuania: The Lithuanian COVIDiet Study. Nutrients.

[90] Năstăsescu V., Mititelu M., Stanciu T.I., Drăgănescu D., Grigore N.D., Udeanu D.I., Stanciu G., Neacșu S.M., Dinu-Pîrvu C.E., Oprea E. (2022). Food Habits and Lifestyle of Romanians in the Context of the COVID-19 Pandemic. Nutrients.

[91] Błaszczyk-Bębenek E., Jagielski P., Bolesławska I., Jagielska A., Nitsch-Osuch A., Kawalec P. (2020). Nutrition Behaviors in Polish Adults before and during COVID-19 Lockdown. Nutrients.

[92] Kosendiak A.A., Wysocki M.P., Krysiński P.P. (2022). Lifestyle, Physical Activity and Dietary Habits of Medical Students of Wroclaw Medical University during the COVID-19 Pandemic. Int. J. Environ. Res. Public Health.

[93] Czenczek-Lewandowska E., Wyszyńska J., Leszczak J., Baran J., Weres A., Mazur A., Lewandowski B. (2021). Health Behaviours of Young Adults during the Outbreak of the COVID-19 Pandemic—A Longitudinal Study. BMC Public Health.

[94] Puścion-Jakubik A., Olechno E., Socha K., Zujko M.E. (2022). Eating Habits during the COVID-19 Pandemic and the Level of Antibodies IgG and FRAP-Experiences of Polish School Staff: A Pilot Study. Foods.

[95] Sekulic M., Stajic D., Jurisic Skevin A., Kocovic A., Zivkovic Zaric R., Djonovic N., Vasiljevic D., Radmanovic B., Spasic M., Janicijevic K. (2022). Lifestyle, Physical Activity, Eating and Hygiene Habits: A Comparative Analysis Before and during the COVID-19 Pandemic in Student Population. Front. Public Health.

[96] Sulejmani E., Hyseni A., Xhabiri G., Rodríguez-Pérez C. (2021). Relationship in Dietary Habits Variations during COVID-19 Lockdown in Kosovo: The COVIDiet Study. Appetite.

[97] Skurvydas A., Lisinskiene A., Lochbaum M., Majauskiene D., Valanciene D., Dadeliene R., Fatkulina N., Sarkauskiene A. (2021). Did COVID-19 Pandemic Change People’s Physical Activity Distribution, Eating, and Alcohol Consumption Habits as Well as Body Mass Index?. Int. J. Environ. Res. Public Health.

[98] Kowalczuk I., Gębski J. (2021). Impact of Fear of Contracting COVID-19 and Complying with the Rules of Isolation on Nutritional Behaviors of Polish Adults. Int. J. Environ. Res. Public Health.

[99] Sińska B., Jaworski M., Panczyk M., Traczyk I., Kucharska A. (2021). The Role of Resilience and Basic Hope in the Adherence to Dietary Recommendations in the Polish Population during the COVID-19 Pandemic. Nutrients.

[100] Zielińska M., Łuszczki E., Bartosiewicz A., Wyszyńska J., Dereń K. (2021). The Prevalence of “Food Addiction” during the COVID-19 Pandemic Measured Using the Yale Food Addiction Scale 2.0 (YFAS 2.0) among the Adult Population of Poland. Nutrients.

[101] Gryszczyńska B., Budzyń M., Grupińska J., Kasprzak M.P., Gryszczyńska A. (2022). Nutritional Behaviors, Vitamin Supplementation and Physical Activity among Polish Adults during the COVID-19 Pandemic. Nutrients.

[102] Smirnova D., Syunyakov T., Pavlichenko A., Bragin D., Fedotov I., Filatova V., Ignatenko Y., Kuvshinova N., Prokopenko E., Romanov D. (2021). Interactions between Anxiety Levels and Life Habits Changes in General Population during the Pandemic Lockdown: Decreased Physical Activity, Falling Asleep Late and Internet Browsing about COVID-19 Are Risk Factors for Anxiety, Whereas Social Media Use Is Not. Psychiatr. Danub..

[103] Hamulka J., Jeruszka-Bielak M., Górnicka M., Drywień M.E., Zielinska-Pukos M.A. (2020). Dietary Supplements during COVID-19 Outbreak. Results of Google Trends Analysis Supported by PLifeCOVID-19 Online Studies. Nutrients.

[104] Hoffmann K., Paczkowska A., Bońka A., Michalak M., Bryl W., Kopciuch D., Zaprutko T., Ratajczak P., Nowakowska E., Kus K. (2022). Assessment of the Impact of the COVID-19 Pandemic on the Pro-Health Behavior of Poles. Int. J. Environ. Res. Public Health.

[105] Modrzejewska J., Modrzejewska A., Czepczor-Bernat K., Matusik P. (2022). The Role of Body Mass Index, Healthy Eating-Related Apps and Educational Activities on Eating Motives and Behaviours among Women during the COVID-19 Pandemic: A Cross Sectional Study. PLoS ONE.

[106] Jodczyk A.M., Gruba G., Sikora Z., Kasiak P.S., Gębarowska J., Adamczyk N., Mamcarz A., Śliż D. (2021). PaLS Study: How Has the COVID-19 Pandemic Influenced Physical Activity and Nutrition? Observations a Year after the Outbreak of the Pandemic. Int. J. Environ. Res. Public Health.

[107] Kupcewicz E., Rachubińska K., Gaworska-Krzemińska A., Andruszkiewicz A., Kuźmicz I., Kozieł D., Grochans E. (2022). Health Behaviours among Nursing Students in Poland during the COVID-19 Pandemic. Nutrients.

[108] Szczepańska E., Janota B., Mąkosza K., Zięba N., Wlazło M., Czapla M. (2022). Consumption of Selected Groups of Food Products by Medical and Non-Medical Students during the COVID-19 Pandemic. Rocz. Panstw. Zakl. Hig..

[109] Larsson K., Onell C., Edlund K., Källberg H., Holm L.W., Sundberg T., Skillgate E. (2022). Lifestyle Behaviors in Swedish University Students before and during the First Six Months of the COVID-19 Pandemic: A Cohort Study. BMC Public Health.

[110] Giacalone D., Frøst M.B., Rodríguez-Pérez C. (2020). Reported Changes in Dietary Habits during the COVID-19 Lockdown in the Danish Population: The Danish COVIDiet Study. Front. Nutr..

[111] Bemanian M., Mæland S., Blomhoff R., Rabben Å.K., Arnesen E.K., Skogen J.C., Fadnes L.T. (2020). Emotional Eating in Relation to Worries and Psychological Distress Amid the COVID-19 Pandemic: A Population-Based Survey on Adults in Norway. Int. J. Environ. Res. Public Health.

[112] Huber B.C., Steffen J., Schlichtiger J., Brunner S. (2021). Altered Nutrition Behavior during COVID-19 Pandemic Lockdown in Young Adults. Eur. J. Nutr..

[113] Palmer K., Bschaden A., Stroebele-Benschop N. (2021). Changes in Lifestyle, Diet, and Body Weight during the First COVID-19 “lockdown” in a Student Sample. Appetite.

[114] Bühlmeier J., Frölich S., Ludwig C., Knoll-Pientka N., Schmidt B., Föcker M., Libuda L. (2022). Changes in Patterns of Eating Habits and Food Intake during the First German COVID-19 Lockdown: Results of a Cross-Sectional Online Survey. Eur. J. Nutr..

[115] Garcia A., Higgs S., Lluch A., Darcel N., Davidenko O. (2021). Associations between Perceived Social Eating Norms and Initiation and Maintenance of Changes in Dietary Habits during the First COVID-19 Lockdown in France. Foods.

[116] Rolland B., Haesebaert F., Zante E., Benyamina A., Haesebaert J., Franck N. (2020). Global Changes and Factors of Increase in Caloric/Salty Food Intake, Screen Use, and Substance Use during the Early COVID-19 Containment Phase in the General Population in France: Survey Study. JMIR Public Health Surveill..

[117] Marty L., de Lauzon-Guillain B., Labesse M., Nicklaus S. (2021). Food Choice Motives and the Nutritional Quality of Diet during the COVID-19 Lockdown in France. Appetite.

[118] Sarda B., Delamaire C., Serry A.-J., Ducrot P. (2022). Changes in Home Cooking and Culinary Practices among the French Population during the COVID-19 Lockdown. Appetite.

[119] Tavolacci M.-P., Ladner J., Déchelotte P. (2021). Sharp Increase in Eating Disorders among University Students since the COVID-19 Pandemic. Nutrients.

[120] Poelman M.P., Gillebaart M., Schlinkert C., Dijkstra S.C., Derksen E., Mensink F., Hermans R.C.J., Aardening P., de Ridder D., de Vet E. (2021). Eating Behavior and Food Purchases during the COVID-19 Lockdown: A Cross-Sectional Study among Adults in the Netherlands. Appetite.

[121] Mertens E., Deriemaeker P., Van Beneden K. (2021). Adjustments in Food Choices and Physical Activity during Lockdown by Flemish Adults. Nutrients.

[122] Drieskens S., Berger N., Vandevijvere S., Gisle L., Braekman E., Charafeddine R., De Ridder K., Demarest S. (2021). Short-Term Impact of the COVID-19 Confinement Measures on Health Behaviours and Weight Gain among Adults in Belgium. Arch. Public Health.

[123] Visser M., Schaap L.A., Wijnhoven H.A.H. (2020). Self-Reported Impact of the COVID-19 Pandemic on Nutrition and Physical Activity Behaviour in Dutch Older Adults Living Independently. Nutrients.

[124] Vandevijvere S., De Ridder K., Drieskens S., Charafeddine R., Berete F., Demarest S. (2021). Food Insecurity and Its Association with Changes in Nutritional Habits among Adults during the COVID-19 Confinement Measures in Belgium. Public Health Nutr..

[125] Dijksterhuis G.B., van Bergen G., de Wijk R.A., Zandstra E.H., Kaneko D., Vingerhoeds M. (2022). Exploring Impact on Eating Behaviour, Exercise and Well-Being during COVID-19 Restrictions in the Netherlands. Appetite.

[126] Kass L., Desai T., Sullivan K., Muniz D., Wells A. (2021). Changes to Physical Activity, Sitting Time, Eating Behaviours and Barriers to Exercise during the First COVID-19 “Lockdown” in an English Cohort. Int. J. Environ. Res. Public Health.

[127] Ogundijo D.A., Tas A.A., Onarinde B.A. (2021). Exploring the Impact of COVID-19 Pandemic on Eating and Purchasing Behaviours of People Living in England. Nutrients.

[128] Buckland N.J., Swinnerton L.F., Ng K., Price M., Wilkinson L.L., Myers A., Dalton M. (2021). Susceptibility to Increased High Energy Dense Sweet and Savoury Food Intake in Response to the COVID-19 Lockdown: The Role of Craving Control and Acceptance Coping Strategies. Appetite.

[129] Robinson E., Boyland E., Chisholm A., Harrold J., Maloney N.G., Marty L., Mead B.R., Noonan R., Hardman C.A. (2021). Obesity, Eating Behavior and Physical Activity during COVID-19 Lockdown: A Study of UK Adults. Appetite.

[130] Theobald C., White A. (2021). British Nutrition Foundation Healthy Eating Week 2020—Insights into the Effect of COVID-19 on Eating and Activity Habits of Adults and Children in the UK. Nutr. Bull..

[131] Baceviciene M., Jankauskiene R. (2021). Changes in Sociocultural Attitudes towards Appearance, Body Image, Eating Attitudes and Behaviours, Physical Activity, and Quality of Life in Students before and during COVID-19 Lockdown. Appetite.

[132] Herle M., Smith A.D., Bu F., Steptoe A., Fancourt D. (2021). Trajectories of Eating Behavior during COVID-19 Lockdown: Longitudinal Analyses of 22,374 Adults. Clin. Nutr. ESPEN.

[133] Vu T.-H.T., Rydland K.J., Achenbach C.J., Van Horn L., Cornelis M.C. (2021). Dietary Behaviors and Incident COVID-19 in the UK Biobank. Nutrients.

[134] Yazıcı D., Fersahoğlu M.M., Fersahoğlu T., Bulut N.E., Çiğiltepe H., Çeler Ö., Sancak S., Sulu C., Durcan E., Şahin S. (2022). Status of Weight Change, Lifestyle Behaviors, Depression, Anxiety, and Diabetes Mellitus in a Cohort with Obesity during the COVID-19 Lockdown: Turk-Com Study Group. Obes. Facts.

[135] Grabia M., Markiewicz-Żukowska R., Puścion-Jakubik A., Bielecka J., Nowakowski P., Gromkowska-Kępka K., Mielcarek K., Socha K. (2020). The Nutritional and Health Effects of the COVID-19 Pandemic on Patients with Diabetes Mellitus. Nutrients.

[136] Ruiz-Roso M.B., Knott-Torcal C., Matilla-Escalante D.C., Garcimartín A., Sampedro-Nuñez M.A., Dávalos A., Marazuela M. (2020). COVID-19 Lockdown and Changes of the Dietary Pattern and Physical Activity Habits in a Cohort of Patients with Type 2 Diabetes Mellitus. Nutrients.

[137] de Luis Román D.A., Izaola O., Primo Martín D., Gómez Hoyos E., Torres Torres B., López Gómez J.J. (2020). Effect of Lockdown for COVID-19 on Self-Reported Body Weight Gain in a Sample of Obese Patients. Nutr. Hosp..

[138] Bellicha A., Lassen P.B., Poitou C., Genser L., Marchelli F., Aron-Wisnewsky J., Ciangura C., Jacques F., Moreau P., Clément K. (2022). Effect of COVID-19 Lockdowns on Physical Activity, Eating Behavior, Body Weight and Psychological Outcomes in a Post-Bariatric Cohort. Obes. Surg..

[139] Conceição E., de Lourdes M., Ramalho S., Félix S., Pinto-Bastos A., Vaz A.R. (2021). Eating Behaviors and Weight Outcomes in Bariatric Surgery Patients amidst COVID-19. Surg. Obes. Relat. Dis..

[140] Madalı B., Alkan Ş.B., Örs E.D., Ayrancı M., Taşkın H., Kara H.H. (2021). Emotional Eating Behaviors during the COVID-19 Pandemic: A Cross-Sectional Study. Clin. Nutr. ESPEN.

[141] Ramalho S.M., Trovisqueira A., de Lourdes M., Gonçalves S., Ribeiro I., Vaz A.R., Machado P.P.P., Conceição E. (2022). The Impact of COVID-19 Lockdown on Disordered Eating Behaviors: The Mediation Role of Psychological Distress. Eat. Weight. Disord..

[142] McAtamney K., Mantzios M., Egan H., Wallis D.J. (2021). Emotional Eating during COVID-19 in the United Kingdom: Exploring the Roles of Alexithymia and Emotion Dysregulation. Appetite.

[143] Tavolacci M.-P., Ladner J., Dechelotte P. (2021). COVID-19 Pandemic and Eating Disorders among University Students. Nutrients.

[144] Cecchetto C., Aiello M., Gentili C., Ionta S., Osimo S.A. (2021). Increased Emotional Eating during COVID-19 Associated with Lockdown, Psychological and Social Distress. Appetite.

[145] Termorshuizen J.D., Watson H.J., Thornton L.M., Borg S., Flatt R.E., MacDermod C.M., Harper L.E., van Furth E.F., Peat C.M., Bulik C.M. (2020). Early Impact of COVID-19 on Individuals with Self-Reported Eating Disorders: A Survey of ~1000 Individuals in the United States and the Netherlands. Int. J. Eat. Disord..

[146] Castellini G., Cassioli E., Rossi E., Innocenti M., Gironi V., Sanfilippo G., Felciai F., Monteleone A.M., Ricca V. (2020). The Impact of COVID-19 Epidemic on Eating Disorders: A Longitudinal Observation of Pre versus Post Psychopathological Features in a Sample of Patients with Eating Disorders and a Group of Healthy Controls. Int. J. Eat. Disord..

[147] McCombie C., Austin A., Dalton B., Lawrence V., Schmidt U. (2020). “Now It’s Just Old Habits and Misery”-Understanding the Impact of the COVID-19 Pandemic on People with Current or Life-Time Eating Disorders: A Qualitative Study. Front. Psychiatry.

